# Prevalencia y severidad de fluorosis dental y factores ambientales y conductuales asociados en niños de una población rural

**DOI:** 10.21142/2523-2754-1402-2026-284

**Published:** 2026-04-04

**Authors:** Ana Juliet Rodriguez-Landauro, Obert Marín-Sánchez, Olegario Marín-Machuca, Katherine Jeanette Campos-Campos

**Affiliations:** 1 EE. SS. Puerta de Entrada San Bartolomé, Red de Salud Huarochirí, Ministerio de Salud del Perú. Cirujano Dentista, Residente de Odontopediatría, Universidad Científica del Sur. Lima, Perú. ana.rodriguez8@unmsm.edu.pe EE. SS. Puerta de Entrada San Bartolomé Red de Salud Huarochirí Ministerio de Salud del Perú ana.rodriguez8@unmsm.edu.pe; 2 Departamento Académico de Microbiología Médica, Facultad de Medicina, Universidad Nacional Mayor de San Marcos. Lima, Perú omarins@unmsm.edu.pe Universidad Nacional Mayor de San Marcos Departamento Académico de Microbiología Médica Facultad de Medicina Universidad Nacional Mayor de San Marcos Lima Peru omarins@unmsm.edu.pe; 3 Departamento Académico de Ciencias Alimentarias, Facultad de Oceanografía, Pesquería, Ciencias Alimentarias y Acuicultura, Universidad Nacional Federico Villarreal. Lima, Perú. omarin@unfv.edu.pe Universidad Nacional Federico Villarreal Departamento Académico de Ciencias Alimentarias Facultad de Oceanografía, Pesquería, Ciencias Alimentarias y Acuicultura Universidad Nacional Federico Villarreal Lima Peru omarin@unfv.edu.pe; 4 Especialidad en Odontopediatría, Facultad de Estomatología, Universidad Científica del Sur. Lima, Perú. kcampos@cientifica.edu.pe Universidad Científica del Sur Especialidad en Odontopediatría Facultad de Estomatología Universidad Científica del Sur Lima Peru kcampos@cientifica.edu.pe

**Keywords:** fluorosis dental, factores de riesgo, cepillado dental, agua potable, dental fluorosis, risk factors, toothbrushing, drinking water

## Abstract

**Introducción:**

La fluorosis dental es una alteración del esmalte originada por una exposición excesiva al fluoruro durante la amelogénesis. Su desarrollo obedece a una interacción multifactorial entre condiciones ambientales, conductuales y biológicas. En comunidades rurales, la falta de supervisión del cepillado infantil y el uso inadecuado de dentífricos fluorados pueden incrementar el riesgo, incluso con niveles bajos de fluoruro en el agua.

**Objetivo:**

Determinar la asociación entre factores ambientales y conductuales con la presencia y severidad de fluorosis dental en niños de 6 a 12 años del distrito San Bartolomé, provincia de Huarochirí, Departamento de Lima.

**Métodos:**

Se realizó un estudio descriptivo y transversal en una muestra censal de 110 escolares. La fluorosis dental se evaluó clínicamente mediante el índice de Thylstrup y Fejerskov (TF). Se analizaron muestras de agua de consumo para determinar la concentración de fluoruro (ppm) y se aplicaron cuestionarios estructurados a los cuidadores para identificar prácticas de higiene oral. El análisis estadístico incluyó pruebas exactas de Fisher y regresión logística bivariada.

**Resultados:**

La prevalencia de fluorosis dental fue del 93,6% y predominaron los grados TF2 (23.6 %), TF3 (18.2 %) y TF5 (17.3 %). El riesgo de fluorosis se asoció significativamente con la edad del niño (p = 0,0004), la edad de inicio del cepillado (p = 0,0269), la ingesta frecuente de pasta dental (p = 0,0010), el uso de pasta de adulto (p = 0,0018) y la cantidad de dentífrico empleada (p < 0,0001). Los modelos de regresión indicaron mayor severidad en niños de 10 y 12 años y una disminución del riesgo en quienes nunca ingerían pasta (OR = 0,015; p = 0,0006) o utilizaban cantidades mínimas de dentífrico (OR = 0,001; p < 0,0001). Las concentraciones de fluoruro en el agua variaron entre 0,13 y 0,36 ppm, por debajo del límite recomendado por la OMS.

**Conclusión:**

Los factores conductuales -inicio precoz del cepillado, uso de pasta dental de adulto, cantidad excesiva e ingesta del dentífrico- se asocian con la alta prevalencia y severidad de la fluorosis dental en esta población, más que la exposición ambiental al fluoruro. Se recomienda fortalecer la educación parental sobre el uso racional de pastas fluoradas y la supervisión del cepillado infantil durante los primeros años de vida.

## Abstract (en)

**Introduction:**

Dental fluorosis is an enamel defect resulting from excessive fluoride exposure during amelogenesis. Its development arises from a multifactorial interaction between environmental, behavioral, and biological factors. In rural communities, lack of supervision during children’s toothbrushing and inappropriate use of fluoride toothpaste may increase the risk, even when water fluoride levels are low.

**Objective:**

To determine the association between environmental and behavioral factors with the presence and severity of dental fluorosis in children aged 6 to 12 years from the district of San Bartolomé, province of Huarochirí, Department of Lima.

**Methods:**

A descriptive, cross-sectional study was conducted in a census sample of 110 schoolchildren. Dental fluorosis was clinically assessed using the Thylstrup and Fejerskov (TF) index. Water samples were analyzed to determine fluoride concentration (ppm), and structured questionnaires were administered to caregivers to evaluate oral hygiene practices. Statistical analysis included Fisher’s exact tests and bivariate logistic regression.

**Results:**

The prevalence of dental fluorosis was 93.6%, with TF2 (23.6%), TF3 (18.2%), and TF5 (17.3%) being the most frequent categories. The risk of fluorosis was significantly associated with age (p = 0.0004), age at brushing initiation (p = 0.0269), frequent toothpaste ingestion (p = 0.0010), use of adult toothpaste (p = 0.0018), and amount of toothpaste used (p < 0.0001). Regression models indicated greater severity among children aged 10 and 12 years, and a reduced risk among those who never ingested toothpaste (OR = 0.015; p = 0.0006) or used minimal amounts (OR = 0.001; p < 0.0001). Fluoride concentrations in drinking water ranged from 0.13 to 0.36 ppm, below the WHO recommended limit.

**Conclusion:**

Behavioral factors-such as early initiation of brushing, use of adult toothpaste, excessive application, and ingestion of dentifrice-account for the high prevalence and severity of dental fluorosis in this population, rather than environmental fluoride exposure. Strengthening parental education on the rational use of fluoridated toothpaste and supervised brushing during early childhood is recommended.

## INTRODUCCIÓN

La fluorosis dental (FD) es un trastorno del desarrollo del esmalte que ocurre como resultado de la ingesta excesiva de fluoruro durante la odontogénesis. Se manifiesta clínicamente con opacidades blancas, estrías, manchas marrones y, en los casos más graves, con pérdida estructural del esmalte, fracturas y mayor susceptibilidad a la caries dental ^1^. El carácter irreversible de estas alteraciones y su origen en etapas tempranas de la vida convierten a la FD en un marcador de exposición ambiental y conductual al fluoruro ^2^.

Históricamente, los primeros reportes sobre la FD se remontan a inicios del siglo XX, cuando G. V. Black y F. McKay describieron, en Colorado (EE. UU.), el fenómeno de los “dientes manchados” en comunidades con aguas subterráneas de alta concentración de fluoruro. Estos hallazgos dieron origen a la denominada “paradoja del fluoruro”: el mismo elemento que producía un defecto estético se asociaba con una notable disminución de la caries dental ^3^. Posteriormente, los estudios de H. Trendley Dean en la década de 1940 establecieron una relación dosis-respuesta entre concentración de fluoruro en agua, prevalencia de fluorosis y reducción de caries, definiendo 1 mg/L como la concentración “óptima” para maximizar beneficios y minimizar riesgos ^4^.

Desde entonces, la fluorización del agua potable fue reconocida como una de las intervenciones de salud pública más costo-efectivas del siglo XX ^5^. Sin embargo, en regiones con niveles naturalmente elevados de fluoruro -como India, China, África y varios países de América Latina- la fluorosis adquirió carácter endémico y se transformó en un problema de salud pública de gran magnitud ^6^.

El estudio de la FD ha pasado de un enfoque epidemiológico a uno más integral, que abarca la comprensión de los mecanismos moleculares de la amelogénesis alterada, el papel de factores modificadores como la nutrición y la genética, así como sus repercusiones psicosociales en la calidad de vida ^7^^-^^9^. La FD trasciende el ámbito odontológico y se configura como un desafío para la salud pública. En su forma leve, puede considerarse un efecto colateral tolerable dada la protección anticaries que ofrece la exposición controlada al fluoruro ^10^. No obstante, en grados moderados y severos, afecta la estética dental, la autoestima y la calidad de vida de los pacientes, lo que genera estigmatización social, sobre todo en niños y adolescentes ^11^.

La prevalencia mundial de fluorosis varía entre el 7,7% y el 80,7% en zonas con agua fluorada, mientras que en áreas con exposición a otras fuentes fluctúa entre el 2,9% y el 42% ^12^. En América Latina, la evidencia disponible es fragmentaria, aunque países como México, Brasil y Colombia han reportado prevalencias crecientes en escolares. En Colombia, por ejemplo, los estudios nacionales de salud bucal (ENSAB) han mostrado un aumento sostenido de la FD desde finales del siglo XX ^13^.

La fluorosis dental no solo representa un marcador visible de exposición excesiva, sino también una señal de alerta para la salud sistémica y las inequidades sociales, dado que afecta con mayor intensidad a comunidades rurales pobres con limitado acceso a servicios odontológicos estéticos y preventivos ^14^. Aún con la amplia evidencia disponible sobre la FD, persisten importantes vacíos de conocimiento. Primero, existe una falta de consenso sobre los límites seguros de exposición al fluoruro. La Organización Mundial de la Salud (OMS) establece 1,5 mg/L como concentración máxima en agua potable, mientras que en Estados Unidos y Canadá el nivel “óptimo” es 0,7 mg/L ^15^. Sin embargo, estudios recientes han documentado prevalencia significativa de fluorosis incluso con concentraciones menores a 1 mg/L ^16^^,^^17^.

Persisten interrogantes sobre la interacción entre factores ambientales (agua, alimentos, contaminantes industriales) y conductuales (uso de dentífricos fluorados, suplementos), así como el papel modulador de variables nutricionales y genéticas ^18^. Por lo tanto, el propósito de este estudio fue determinar la prevalencia y severidad de la fluorosis dental en niños de 6 a 12 años de un distrito rural y su asociación con factores ambientales y conductuales. 

## MATERIALES Y MÉTODOS

### Diseño del estudio

Estudio observacional, descriptivo, prospectivo y de corte transversal, orientado a identificar factores ambientales y conductuales asociados a la fluorosis dental en población infantil rural. 

### Población y muestra

La población de estudio estuvo conformada por todos los niños de 6 a 12 años, residentes en el distrito de San Bartolomé y sus anexos, provincia de Huarochirí (Lima, Perú). Se realizó un muestreo censal, que incluyó a la totalidad de la población elegible. Fueron evaluados 110 niños, cuyos padres firmaron el consentimiento informado y que presentaban dientes permanentes erupcionados. Se excluyó a los niños con enfermedades sistémicas, síndromes genéticos y aquellos que presentaban restauraciones estéticas en las superficies vestibulares de los dientes anteriores permanentes. 

### Recolección de los datos

La fluorosis dental fue medida con el índice de Thylstrup y Fejerskov (TF), categorizado de TF0 a TF9. La variable ambiental incluyó la concentración de fluoruro en el agua potable proveniente de pozos y conexiones domiciliaras. Las variables conductuales incluyeron responder un cuestionario sobre el uso de dentífrico, la edad del inicio del cepillado, la supervisión del cepillado por un adulto y la cantidad de pasta dental fluorada utilizada. Se llevaron a cabo sesiones informativas dirigidas a niños y padres, en las que se explicaron la naturaleza y los objetivos del estudio, y se puso énfasis en el carácter voluntario de la participación. 

### Evaluación de FD 

Se realizó una evaluación clínica en campo, bajo luz natural, para identificar signos de fluorosis mediante la aplicación del índice TF. La investigadora fue previamente capacitada y calibrada con un especialista, y se obtuvo un índice de Kappa mayor a 0,8. Se tomaron fotografías intraorales estandarizadas para confirmar el diagnóstico clínico, evaluadas posteriormente por un especialista calibrado también previamente. Todos los datos fueron consignados en una ficha técnica previamente validada y, posteriormente, procesados para el análisis estadístico.

### Medición de variables conductuales

Los padres firmaron el consentimiento informado y respondieron un cuestionario estructurado sobre prácticas de higiene oral durante la primera infancia.

### Medición del flúor en el agua

Se recolectaron muestras de agua de pozos y conexiones domiciliarias en las cuatro localidades. El análisis de fluoruro fue realizado por el laboratorio acreditado Delta Lab (registro INACAL N.° LE-077), aplicando el método SPADNS (SMEWW-APHA-AWWA-WEF 4500-F D 23.ª Ed., 2017).

### Análisis estadístico

Los datos fueron ingresados en una base de Excel 2003 y analizados con el *software* R versión 4.5.0. Se realizó estadística descriptiva para resumir las variables cuantitativas (medidas de tendencia central y dispersión) y cualitativas (frecuencias absolutas y relativas). Para el análisis bivariado, se calcularon razones de momios (OR crudos) con sus intervalos de confianza al 95%, utilizando regresión logística binaria. En los casos de separación completa o escasez de datos en algunas categorías, se aplicó regresión logística penalizada de Firth para garantizar la estabilidad de las estimaciones. Posteriormente, se ajustó un modelo multivariable que incluyó las variables con relevancia clínica y aquellas con significancia estadística en el análisis bivariado. Los resultados se expresaron como *odds ratio* ajustados (ORa) con sus intervalos de confianza del 95%. Se consideró un nivel de significancia de p < 0,05 para todas las pruebas.

### Consideraciones éticas

El estudio fue aprobado por el Comité Institucional de Ética en Investigación del Instituto de Medicina Tropical Daniel Alcides Carrión, de la Facultad de Medicina de la Universidad Nacional Mayor de San Marcos, antes de iniciar la investigación, mediante constancia de aprobación CIEI-2018-029. Todos los procedimientos se llevaron a cabo conforme a los principios de la Declaración de Helsinki. La participación de los menores fue voluntaria, mediada por el consentimiento informado firmado por los tutores legales, y se garantizó en todo momento la confidencialidad y el anonimato de la información.

## RESULTADOS

Se evaluaron 110 niños entre 6 y 12 años (X-: 9,8 años) del distrito estudiado. La muestra estuvo conformada por 59 niñas (53,6%) y 51 niños (46,4%). En cuanto a la distribución etaria, el grupo más numeroso correspondió a los de 12 años (32,7%), seguido de los de 8 años (17,3%) y 11 años (14,5%). La prevalencia global de fluorosis dental fue del 93,6%, con predominio de los grados TF2 (23,6%), TF3 (18,2%) y TF5 (17,3%). La mayoría de los participantes procedía de zonas rurales (66.4%), mientras que un 20% eran de áreas urbanas. Respecto de los hábitos de higiene, el 96,4% consumía agua proveniente de conexión domiciliaria. La mayoría reportó haber iniciado el cepillado antes de los 3 años (88,2%) y utilizar pasta dental de adulto en algunas ocasiones (53,6%) o siempre (36,4%). En cuanto a la cantidad de dentífrico, el 55,5% usaba el tamaño de una arveja, mientras que el 40% aplicaba el cepillo completo. La ingesta de pasta dental fue reportada como “casi siempre” por el 40% de los niños y “pocas veces” por el 52,7% (Tabla 1).

**Tabla 1:** Distribución de las características de la muestra

Variable	Categoría	n (%)
Sexo	Femenino	59 (53.6%)
	Masculino	51 (46.4%)
Edad	6	12 (10.9%)
	7	5 (4.5%)
	8	19 (17.3%)
	9	10 (9.1%)
	10	12 (10.9%)
	11	16 (14.5%)
	12	36 (32.7%)
Fluorosis dental	TF0	7 (6.4%)
	TF1	18 (16.4%)
	TF2	26 (23.6%)
	TF3	20 (18.2%)
	TF4	11 (10.0%)
	TF5	19 (17.3%)
Procedencia	Rural	73 (66.4%)
	Urbana	22 (20.0%)
	Otro	4 (3.6%)
	No especificado	11 (10.0%)
Tipo de agua	Conexión domiciliaria	106 (96.4%)
	Riachuelo	4 (3.6%)
Uso de pasta de adulto	Algunas veces	59 (53.6%)
	Nunca lo ha usado	11 (10.0%)
	Siempre	40 (36.4%)
Edad de inicio cepillado	Antes de los 3 años	97 (88.2%)
	Después de los 3 años	13 (11.8%)
Cantidad de pasta dental	Cepillo completo	44 (40.0%)
	Grano de arroz	5 (4.5%)
	Tamaño arveja	61 (55.5%)
Ingesta de pasta dental	Casi siempre	44 (40.0%)
	Nunca lo ha hecho	8 (7.3%)
	Pocas veces	58 (52.7%)

TF0: sin fluorosis

TF1: líneas blancas suaves.

TF2: pequeñas áreas opacas.

TF3: áreas difusas de opacidad y líneas blancas.

TF4: toda la superficie presenta opacidad (aspecto de “piedra caliza blanca”).

TF5: superficie opaca con depresiones redondeadas menores a 2 mm de diámetro.

La distribución de la severidad de fluorosis dental según edad se muestra en la Tabla 2. La categoría más frecuente fue TF2 (27,7%), seguida por TF3 (21,3%) y TF5 (20,2%). Se observó una tendencia al incremento de la severidad con la edad. A los 6 y 7 años, predominaron los casos leves (TF1: 100%), mientras que en los grupos de 11 y 12 años fueron más frecuentes los grados moderados y severos (TF3-TF5). En particular, los niños de 11 años presentaron una proporción elevada de TF5 (42,9%), y los de 12 años mostraron la mayor dispersión de grados, con TF3 (30,6%) y TF5 (25%) como los más prevalentes. Estos resultados evidencian un patrón progresivo de incremento en la severidad de fluorosis con la edad.

**Tabla 2:** Frecuencia de la fluorosis dental de acuerdo con la severidad y la edad

Edad	TF1	TF2	TF3	TF4	TF5	Total
6	3 (100%)					3
7	1 (100%)					1
8	3 (16.7%)	8 (44.4%)	3 (16.7%)	3 (16.7%)	1 (5.6%)	18
9		5 (50%)	2 (20%)	1 (10%)	2 (20%)	10
10	3 (25%)	5 (41.7%)	3 (25%)		1 (8.3%)	12
11	3 (21.4%)	3 (21.4%)	1 (7.1%)	1 (7.1%)	6 (42.9%)	14
12	5 (13.9%)	5 (13.9%)	11 (30.6%)	6 (16.7%)	9 (25%)	36
Total	18 (19.1%)	26 (27.7%)	20 (21.3%)	11 (11.7%)	19 (20.2%)	94

TF1: líneas blancas suaves.

TF2: pequeñas áreas opacas.

TF3: áreas difusas de opacidad y líneas blancas.

TF4: toda la superficie presenta opacidad (aspecto de “piedra caliza blanca”).

TF5: superficie opaca con depresiones redondeadas menores a 2 mm de diámetro.

**Figura 1:**
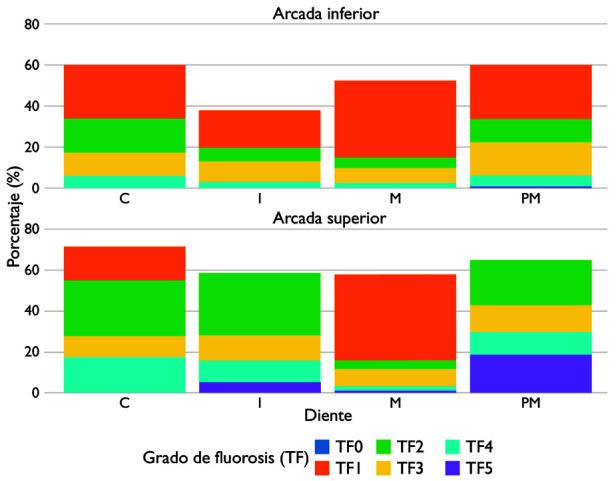
Distribución de los porcentajes de dientes con fluorosis dental según severidad y arco dental

Según el tipo de diente, el grado TF2 fue el diagnóstico más frecuente en los incisivos centrales superiores e inferiores, y en los incisivos laterales inferiores. En cambio, el grado TF3 predominó en los segundos molares permanentes, mientras que el TF2 también fue el más común en los incisivos laterales superiores y en los primeros molares permanentes. En general, se observó una distribución más homogénea de los grados leves y moderados (TF1-TF3) en la arcada superior, mientras que la arcada inferior presentó una mayor concentración de grados severos (TF4-TF5), especialmente en los molares. Las comparaciones entre ambas arcadas mostraron una correlación moderadamente fuerte en la severidad de fluorosis (rho = 0,693; p < 0,001), lo cual indica que la afectación en un arco tiende a corresponder con el grado de afectación en el otro (Figura 1).

El análisis bivariado mostró una asociación estadísticamente significativa entre la presencia de fluorosis dental y las variables edad del niño (p = 0,0004), edad de inicio del cepillado (p = 0,0269), ingesta de pasta dental (p = 0,0010), uso de pasta dental de adulto (p = 0,0018) y cantidad de pasta utilizada (p < 0,0001). En contraste, la procedencia y el tipo de agua no mostraron relación significativa con la presencia de fluorosis (p > 0,05). El sexo presentó una asociación estadísticamente significativa con la presencia de fluorosis en el análisis bivariado (p = 0,014); sin embargo, esta asociación debe interpretarse con cautela dado el desequilibrio en la distribución por celdas -ningún niño de sexo masculino se clasificó en el grupo sin fluorosis (0 de 51)-, lo que genera condiciones de separación cuasi-completa y limita la estabilidad de la estimación^5^. De manera particular, los niños que iniciaron el cepillado antes de los 3 años presentaron una mayor frecuencia de fluorosis (86 de 90; 95,5%), mientras que aquellos que ingerían pasta dental con mayor frecuencia o utilizaban pasta de adulto de forma constante mostraron proporciones notablemente más elevadas de afectación. Asimismo, el uso de una cantidad excesiva de dentífrico (cepillo completo) se asoció de forma muy significativa con la presencia de fluorosis (p < 0,0001) (Tabla 3).

**Tabla 3:** Análisis bivariado entre fluorosis y factores asociados

Variable	Categoría	Fluorosis dental	Fluorosis dental	p
		Presencia	Ausencia	
Sexo	Femenino	47	7	0,0140
	Masculino	47	0	
Edad	6	3	2	0,0004*
	7	1	2	
	8	18	1	
	9	10	0	
	10	12	0	
	11	14	2	
	12	36	0	
Procedencia	Rural	64	3	0,2175
	Urbana	18	2	
	Otro	3	0	
	No especificado	9	2	
Tipo de agua	Conexión domiciliaria	91	7	1,0000
	Riachuelo	3	0	
Edad inicio cepillado	Antes de los 3 años	86	4	0,0269*
	Después de los 3 años	8	3	
Ingesta de pasta dental	Casi siempre	43	0	0,0010*
	Nunca lo ha hecho	4	3	
	Pocas veces	47	4	
Uso de pasta adulto	Algunas veces	49	4	0,0018*
	Nunca lo ha usado	5	3	
	Siempre	40	0	
Cantidad de pasta	Cepillo completo	44	0	0,0000*
	Grano de arroz	0	4	
	Tamaño arveja	50	3	

Test exacto de Fisher (bivariado) * p < 0.05

En el análisis univariable de Firth, luego del ajuste de las principales variables de confusión, se observó que la edad, la ingesta de pasta dental, la edad de inicio del cepillado y la cantidad de pasta utilizada fueron los factores con mayor peso sobre la severidad de la fluorosis dental (Tabla 4). Los niños de 10 y 12 años presentaron un riesgo significativamente mayor de fluorosis severa en comparación con los de 6 años (OR = 17,86; p = 0,0408 y OR = 52,14; p = 0,0039, respectivamente). Asimismo, quienes nunca ingerían pasta dental mostraron una reducción notable en la probabilidad de fluorosis severa (OR = 0,015; p = 0,0006). El inicio del cepillado después de los 3 años también se asoció con una menor severidad (OR = 0,126; p = 0,0149), al igual que el uso de una cantidad mínima de dentífrico (grano de arroz) (OR = 0,001; p < 0,0001). En cambio, el sexo masculino presentó una razón de momios elevada (OR = 15,0; p = 0,0087), lo que sugiere una mayor predisposición a formas más severas de fluorosis. No se encontraron asociaciones significativas con la procedencia ni con el tipo de agua consumida.

**Tabla 4:** Correlación de las características y la severidad de la fluorosis dental

Variable	Categoría	OR_crudo	p_crudo
Sexo	Femenino	1,00 (Ref)	-
	Masculino	15,000 (1,743-1967,164)	0,0087*
Edad	6	1,00 (Ref)	-
	7	0,429 (0,025-5,556)	0,5169
	8	8,810 (0,905-122,457)	0,0607
	9	15,000 (0,929-2280,949)	0,0569
	10	17,857 (1,120-2705,476)	0,0408*
	11	4,143 (0,484-38,191)	0,1875
	12	52,143 (3,409-7799,519)	0,0039*
Procedencia	Rural	1,00 (Ref)	-
	Urbana	0,402 (0,072-2,566)	0,3093
	Otro	0,380 (0,027-54,858)	0,5848
	No especificado	0,206 (0,035-1,375)	0,0969
Tipo de agua consumida antes de los 3 años	Domiciliaria	1,00 (Ref)	-
	Riachuelo	0,574 (0,048-79,897)	0,7377
Edad de inicio del cepillado con pasta dental	Antes de los 3 años	1,00 (Ref)	-
	Después de los 3 años	0,126 (0,026-0,646)	0,0149*
Ingesta de pasta dental	Casi siempre	1,00 (Ref)	-
	Nunca lo ha hecho	0,015 (0,000-0,186)	0,0006*
	Pocas veces	0,121 (0,001-1,188)	0,0741
Uso de pasta dental de adulto	Algunas veces	1,00 (Ref)	-
	Nunca lo ha usado	0,143 (0,026-0,781)	0,0261
	Siempre	7,364 (0,751-986,691)	0,0944
Cantidad de pasta dental antes de los 3 años	Cepillo completo	1,00 (Ref)	-
	Grano de arroz	0,001 (0,000-0,030)	0,0000*
	Tamaño arveja	0,162 (0,001-1,739)	0,1482

Regresión logística penalizada de Firth (modelo univariable) * p < 0.05

## DISCUSIÓN

La prevalencia de fluorosis dental (FD) encontrada en la población infantil del distrito de San Bartolomé y sus anexos fue del 93,6%, una cifra notablemente superior a la descrita en la mayoría de los estudios nacionales e internacionales con concentraciones similares o incluso mayores de fluoruro en el agua potable ^1^^,^^3^^,^^7^^,^^13^. Este hallazgo confirma que la FD no depende exclusivamente del contenido de fluoruro en el agua, sino que obedece a una interacción multifactorial entre condiciones biológicas, conductuales y ambientales ^1^^,^^6^^,^^10^.

Las concentraciones de fluoruro en el agua oscilaron entre 0,13 y 0,36 ppm, valores considerablemente inferiores al límite recomendado por la OMS (1,5 mg/L) ^15^^,^^16^. A pesar de ello, la prevalencia observada fue alta, lo que sugiere una exposición conductual significativa. Los resultados mostraron que la mayoría de los niños inició el cepillado antes de los 3 años, utilizó pastas dentales para adultos y aplicó cantidades excesivas de dentífrico, prácticas asociadas a un mayor riesgo de fluorosis ^17^^,^^19^^,^^20^. En este grupo, la ingesta frecuente de pasta dental y el uso de pasta de adulto presentaron asociaciones altamente significativas con la presencia y severidad de FD, lo que coincide con estudios que estiman que los niños pequeños pueden ingerir entre un 30 % y un 60 % del dentífrico en cada cepillado ^19^^,^^20^.

La exposición temprana y continua al fluoruro durante esta fase crítica interfiere con la maduración del esmalte, lo que genera lesiones más extensas y visibles a medida que los dientes erupcionan ^2^^,^^6^^,^^19^^,^^20^. Además, los dientes que finalizan su mineralización más tardíamente -como los caninos y segundos premolares- permanecen más tiempo expuestos a hábitos inadecuados de cepillado e ingestión de dentífrico, lo que puede explicar la relación directa entre mayor edad y mayor severidad encontrada en este estudio. Este comportamiento ha sido descrito también por Fejerskov y colaboradores ^1^^,^^3^, quienes señalan que la exposición acumulativa durante la amelogénesis amplifica los defectos subsuperficiales del esmalte y condiciona la expresión clínica de fluorosis moderada o severa.

En relación con la distribución anatómica, se observó que los grados leves y moderados (TF1-TF3) fueron los más frecuentes, con predominio en los incisivos superiores y premolares, mientras que los grados severos (TF4-TF5) se presentaron con menor frecuencia. Este patrón concuerda con la literatura que describe una mayor susceptibilidad de los incisivos permanentes debido a su periodo prolongado de mineralización y a la elevada visibilidad durante la amelogénesis, etapa particularmente sensible al exceso de fluoruro ^2^^,^^6^^,^^8^. Las diferencias entre arcadas podrían estar asociadas con variaciones en el tiempo de erupción, la morfología y la exposición salival. La arcada superior, por ejemplo, suele acumular más placa y dentífrico en la zona anterior durante el cepillado, lo que aumenta la exposición local al fluoruro en niños pequeños que aún no controlan la deglución ^6^^,^^17^^,^^19^. Además, la correlación moderadamente fuerte entre ambas arcadas (rho = 0,693; p < 0,001) indica un patrón sistémico de afectación bilateral, coherente con la absorción y distribución uniforme del fluoruro durante la formación del esmalte ^1^^,^^2^^,^^20^.

Desde el punto de vista biológico, la FD se origina por la alteración de la amelogénesis. El fluoruro puede interferir con la actividad de los ameloblastos, modificar la orientación cristalina del esmalte y alterar la degradación proteica mediada por las enzimas MMP-20 y KLK-4, lo cual genera un esmalte subsuperficial poroso y clínicamente opaco ^2^^,^^3^^,^^6^^,^^8^^,^^20^. Además, el estrés oxidativo y la disfunción del retículo endoplásmico inducen apoptosis en los ameloblastos, hecho que agrava los defectos estructurales ^7^^,^^22^.

A nivel genético y nutricional, la susceptibilidad individual frente al fluoruro puede explicarse por polimorfismos en genes relacionados con la formación del esmalte (AMELX, ENAM, MMP20, KLK4), así como por deficiencias de calcio y vitamina D que afectan la mineralización ^6^^,^^10^^,^^21^. La elevada prevalencia observada, aun con bajos niveles ambientales de fluoruro, respalda este modelo multifactorial en el que la exposición temprana y no supervisada a dentífricos fluorados representa el principal factor de riesgo en esta población.

En términos clínicos, la afectación predominó en incisivos superiores y premolares, lo que coincide con reportes previos que señalan que los dientes anteriores y de mayor visibilidad son los más comprometidos ^2^^,^^16^. Aunque la mayoría de los casos correspondió a grados leves o moderados (TF1-TF3), no deben subestimarse sus repercusiones estéticas y psicológicas. Se ha documentado que incluso formas leves de FD pueden impactar la autoestima y la calidad de vida relacionada con la salud oral ^3^^,^^7^^,^^10^.

Este fenómeno refleja la conocida “paradoja del fluoruro”: el mismo elemento que previene la caries dental puede provocar defectos irreversibles del esmalte cuando la exposición excede el umbral biológico de seguridad ^1^^,^^3^^,^^7^. Diversos estudios reportan prevalencias elevadas de FD en comunidades con niveles de fluoruro menores a 1,0 mg/L, lo que sugiere que el límite universal de 1,5 mg/L establecido por la OMS podría no ser adecuado para todas las condiciones geográficas o culturales ^10^^,^^13^^,^^16^^,^^21^.

Desde la perspectiva de salud pública, los resultados subrayan la urgencia de promover el uso racional de pastas fluoradas en la infancia, conforme a las recomendaciones internacionales: cantidad equivalente a un grano de arroz para menores de tres años y del tamaño de un guisante entre tres y seis años, siempre bajo supervisión adulta ^17^^,^^19^^,^^20^. Asimismo, se requieren programas educativos que equilibren los beneficios anticaries del fluoruro con la prevención de la FD, especialmente en comunidades rurales donde la supervisión del cepillado es limitada ^13^^,^^18^.

En conclusión, estos hallazgos indican que la severidad de la fluorosis está fuertemente influenciada por la edad y los hábitos de higiene oral, particularmente por la frecuencia y cantidad de pasta dental utilizada. La fluorosis dental en la población escolar del distrito de San Bartolomé constituye un problema de salud pública de alta prevalencia (93,6%), con predominio de formas leves a moderadas (TF2 y TF3). Los resultados demostraron de manera consistente que la fluorosis se asocia principalmente con factores conductuales de exposición temprana al fluoruro, en especial el inicio del cepillado antes de los 3 años, el uso de pastas dentales con concentración de adulto (≈1450 ppm de F−), la falta de supervisión parental y la ingesta repetida de dentífrico durante el cepillado. Este patrón se asocia el aumento de la severidad observado en los niños de 10 a 12 años, coincidente con el periodo crítico de amelogénesis de incisivos y premolares. 

En este contexto, Saldarriaga ^23^ destaca que el uso de dentífricos con altas concentraciones de fluoruro (≥ 1450 ppm) sin supervisión en menores de tres años incrementa significativamente el riesgo de fluorosis, sobre todo cuando se ingieren cantidades apreciables de pasta. No obstante, Angmar-Månsson y Whitford ^24^ demostraron que factores fisiológicos -como alteraciones en el equilibrio ácido-base o la residencia a gran altitud- pueden modificar la excreción renal y la retención tisular del fluoruro, lo que amplifica sus efectos sobre los ameloblastos incluso con exposiciones moderadas. Los hallazgos sustentan la necesidad de intervenciones preventivas tempranas enfocadas en la educación de los padres y cuidadores sobre el uso racional del dentífrico fluorado y la supervisión estricta del cepillado infantil, para evitar la exposición innecesaria al fluoruro durante la formación del esmalte.

## Fuentes de financiamiento:

El estudio fue autofinanciado por los autores
